# Real-world use of remdesivir for the treatment of patients admitted to Italian hospitals with COVID-19: the nationwide retrospective FADOI-RECOVER study

**DOI:** 10.1186/s12879-023-08422-6

**Published:** 2023-07-08

**Authors:** Filippo Pieralli, Fulvio Pomero, Francesco Dentali, Claudio Norbiato, Tiziana Attardo, Susanna Vicari, Elena Magnani, Maria Antonietta Marzilli, Paola Piccolo, Antonella Valerio, Dario Manfellotto, on behalf of FADOI RECOVER Study Group, Elena Brugiotti, Vincenzo Carella, Martina Coppo, Francesca Ferrando, Marta. Lauritano, Bruno Marchetti, Francesco Vitale, Daniela Dalla Gasperina, Elena Baroni, Andrea Boccatonda, Enrico Giorgini, Teresa Milite, Luca Montaguti, Elisa Cagnoni, Giulia Mogavero, Giovanni Capoccetta, Raffaella De Giovanni, Francesca Martelli, Giulia Guazzini, Alberto Grassi, Laura Romani, Paola Gnerre, Franco Mastroianni, Fabiana D’Onofrio, Sergio Berra, Simona Pozzoli, Flavio Bobbio, Sara Bianco, Azzurra Re, Nicola Liberato, Sara Job, Giancarlo Antonucci, Ombretta Para, Gino Ferrara, Antonietta Giordano, Olga Falco, Roberto Manetti, Carolina Bologna, Sandra Buscaglia, Cristina Oliviero, Maria Amitrano, Valeria Iorio, Loredana Tibullo, Giovanni Ferrari, Micaela Brandolini, Giovanna Leone, Carlo Usai, Noemi Elisabetta Manzoni, Rita Di Stefano, Eusapia Renna

**Affiliations:** 1 Internal Medicine and Intermediate Care Unit, Careggi University Hospital, Florence, Italy; 2 Internal Medicine, Michele and Pietro Ferrero Hospital, Verduno, Cuneo, Italy; 3 Department of Emergency of High-Specialty and Medical Center, ASST-Settelaghi, Varese, Italy; 4 Internal Medicine, Ordine Mauriziano di Torino Hospital, Turin, Italy; 5 Internal Medicine Department, Ospedale di Circolo e Fondazione Macchi, ASST-Settelaghi, Varese, Italy; 6 Internal Medicine, Bentivoglio Hospital, AUSL Bologna, Bentivoglio, Bologna, Italy; 7 Internal Medicine, M. Bufalini Hospital, AUSL Romagna, Cesena, Italy; 8 Internal Medicine Department, SS. Trinità Hospital, Cagliari, Italy; 9 Internal Medicine, Fatebenefratelli Isola Tiberina Hospital, Gemelli Isola, Rome, Italy; 10 Research Department, FADOI Foundation, Piazzale Cadorna, 15, 20123 Milan, Italy; 11 Sub-intensive COVID Internal Medicine Division, Rimini Hospital, Rimini, Italy; 12 Internal Medicine 1 COVID Division, Rimini Hospital, Rimini, Italy; 13 Internal Medicine Department, Acqui Terme Hospital, Acqui Terme, Alessandria, Italy; 14 Internal Medicine Department, Francesco Miulli General Hospital, Acquaviva delle Fonti, Bari, Italy; 15 Internal Medicine Department, Garbagnate Milanese Hospital, Garbagnate Milanese, Milan, Italy; 16 Internal Medicine Department, Maggiore della Carità Hospital, Novara, Italy; 17 Internal Medicine Department, Melegnano Hospital, Melegnano, Milan, Italy; 18 Internal Medicine Department, Galliera Hospital, Genova, Italy; 19 Internal Medicine 1 Department, Careggi University Hospital, Florence, Italy; 20 Internal Medicine Division, Frattamaggiore Hospital, Napoli, Italy; 21 Clinica Medica Department, Sassari Hospital, Sassari, Italy; 22 Internal Medicine Department, Ospedale del Mare ASL Na 1, Naples, Italy; 23 Internal Medicine Department, San Paolo Hospital, Savona, Italy; 24 Internal Medicine Unit, Moscati Hospital, Avellino, Italy; 25 Internal Medicine Department, Broni-Stradella Hospital, Stradella, Pavia, Italy; 26 Internal Medicine Department, Faenza Hospital, Ravenna, Italy; 27 Internal Medicine Department, S.S. Annunziata Hospital, Sassari, Italy; 28 Internal Medicine Department, San Paolo Hospital, Bari, Italy

**Keywords:** Remdesivir, Internal Medicine, COVID-19 pneumonia, Management of COVID-19

## Abstract

**Background:**

Remdesivir is widely used for treatment of SARS-CoV-2 pneumonia. The aim of this study was to evaluate the characteristics of patients with moderate-to-severe COVID-19 treated with remdesivir, and their outcomes during hospitalization.

**Methods:**

This retrospective observational multicenter study included consecutive patients, hospitalized for moderate-to-severe COVID-19 (September 2020—September 2021), who were treated with remdesivir.

**Results:**

One thousand four patients were enrolled, all with onset of symptoms occurring less than 10 days before starting remdesivir; 17% of patients had 4 or more concomitant diseases. Remdesivir was well tolerated, adverse drug reactions (ADRs) being reported in 2.3% of patients. In-hospital death occurred in 80 patients (8.0%). The median timing of the first remdesivir dose was 5 days after symptom onset. The following endpoints did not differ according to the time span from the onset of symptoms to the first dose: length of hospitalization, in-hospital death, composite outcome (in-hospital death and/or endotracheal intubation). Advanced age, number of comorbidities ≥ 4, and severity of respiratory failure at admission were associated with poor in-hospital outcomes.

**Conclusion:**

In a real-world setting, remdesivir proved to be a safe and well-tolerated treatment for moderate-to-severe COVID-19. In patients receiving remdesivir less than 3 or 5 days from the onset of SARS-CoV-2 symptoms, mortality and the need for mechanical ventilation did not differ from the rest of the sample.

## Introduction

On 11 March 2020, a World Health Organization (WHO) declaration recognized the outbreak of SARS CoV2 infection and COVID-19 as a worldwide pandemic. The resulting emergency placed healthcare systems all over the world under enormous pressure. To date, more than 750 million confirmed cases and 6.8 million attributable deaths have been recorded globally, including nearly 200,000 deaths in Italy [1]. After initial uncertainty and concern about the efficacy and safety of drugs empirically used for the treatment of COVID-19 [2], remdesivir was the first antiviral to obtain emergency authorization from the European Medicines Agency (EMA) with a specific indication for the “treatment of COVID-19 disease with pneumonia requiring supplemental oxygen therapy” [3].

Remdesivir is an antinucleotide prodrug (ProTide) which, after activation, acts as an analogue of adenosine triphosphate (ATP) and competes with the natural substrate of ATP for incorporation into nascent RNA chains by SARS-CoV-2 RNA-dependent RNA polymerase, ultimately inhibiting replication of the SARS-CoV-2 virus [4–8].

In most published studies, from randomized clinical trials (RCTs) in different clinical settings to systematic reviews and meta-analyses, remdesivir generally showed reduced time to recovery, with no effect on hard endpoints, such as death and/or the need for endotracheal intubation (ETI) in patients hospitalized with moderate-to-severe COVID-19 [9–11]. A number of studies, including the World Health Organization (WHO)-promoted Solidarity Trial [12, 13], also showed that remdesivir provides benefits in patients with moderate-to-severe COVID-19 requiring oxygen therapy, but not in those requiring high-flow oxygen therapy or mechanical ventilation (invasive or non-invasive) or extracorporeal membrane oxygenation (ECMO). The beneficial effects were observed in hospitalized patients who received remdesivir within 10 days from symptom onset, while no effect was observed in those treated after that time span [10, 12–16].

More recently, the final report of the WHO Solidarity Trial Consortium [17] and meta-analysis of RCTs by the American College of Physicians [18] showed reduced mortality in patients with COVID-19 treated with remdesivir who were on oxygen therapy but not on mechanical ventilation; similar evidence is provided by retrospective studies in real-life settings [19, 20]. The largest study, to date, is a retrospective cohort study including more than 2,300 patients treated with remdesivir, where antiviral treatment was associated with a significant reduction in inpatient mortality, across different degrees of disease severity, compared to controls matched through propensity score and risk-set sampling [21].

Considering the high number of COVID-19 patients managed in Italian Internal Medicine departments and treated with remdesivir, the FADOI (Italian Scientific Society of Internal Medicine) Foundation promoted a retrospective nationwide observational study to evaluate the characteristics of patients with moderate-to-severe COVID-19 treated with this drug, and their clinical outcome during hospitalization; the objective was to provide a real-world complement to the evidence from RCTs conducted in experimental settings.

## Methods

### Study design and setting

FADOI-RECOVER is a retrospective observational study, sponsored, coordinated and run by the FADOI Foundation.

Investigators retrospectively reviewed hospital records of all consecutive patients hospitalized between September 2020 and September 2021 with SARS-CoV-2 infection, treated with remdesivir. SARS-CoV-2 infection’s diagnosis was by real-time reverse transcriptase polymerase chain reaction (RT-PCR) assay on a naso-pharyngeal swab and/or bronchoalveolar lavage, together with radiological documentation of pneumonia. Patients were enrolled at 26 Italian Internal Medicine Units, reasonably representative of the National Health System hospital network as a whole in terms of geographical spread and catchment area.

Patients were selected according to the criteria defined by the Italian Medicines Agency (AIFA) for the use of remdesivir in hospitalized COVID-19 patients, namely:

- Age ≥ 18 years, with pneumonia requiring supplemental oxygen therapy
- Radiologically documented diagnosis of pneumonia
- Onset of symptoms less than 10 days before
- Need for additional low-flow oxygen therapy
- No need for non-invasive mechanical ventilation or high-flow oxygen therapy delivered by high-flow nasal cannula
- No need for invasive mechanical ventilation or ECMO
- Estimated glomerular filtration rate (eGFR) > 30 mL/min
- Normal values of alanine aminotransferase (ALT), or < 5 times the upper limit of normal at baseline
- Normal values of conjugated bilirubin, alkaline phosphatase or International Normalized Ratio (INR).

The study, following approval of the protocol (FADOI-RECOVER, protocol code FADOI.02–2021) by local Ethics Committees, was carried out in full compliance with the Declaration of Helsinki and the principles of Good Clinical Practice.

### Data collection and objectives

Demographic and clinical characteristics were recorded for each patient, as well as laboratory variables and drug therapies at the time of admission and during the hospital stay. Recorded variables included the SO_2_/FiO_2_ ratio, a non-invasive parameter to estimate the severity of respiratory failure in COVID-19-associated ARDS [22].

Other parameters/events recorded were: time since symptom onset, need for ETI, drug-related adverse reactions, length of hospital stay, and in-hospital death.

The main purpose of the study was to describe, in a real-world setting, the clinical characteristics of patients admitted for COVID-19 pneumonia and treated with remdesivir as indicated by formal AIFA criteria. A second aim was to examine how long after the onset of symptoms remdesivir was first administered, evaluating any correlation between timing of the initial dose and clinically relevant outcome measures, such as respiratory function deterioration during hospitalization, length of hospital stay, need for ETI/ECMO, and in-hospital death. Finally, we aimed to evaluate the safety profile of remdesivir by recording adverse drug reactions (ADRs).

### Statistical analysis

Continuous variables were expressed as mean ± standard deviation (SD) or median and interquartile range (IQR) values, while categorical data were expressed as proportions and percentages. The Student t-test and one-way analysis of variance (ANOVA) models were used for the comparison of continuous normally distributed variables, and the Mann–Whitney U test for continuous variables that were not normally distributed. The χ-square test or Fisher’s exact test, when appropriate, were used for the comparison of categorical variables. Risk factors for the occurrence of in-hospital death and the composite outcome measure of in-hospital death and/or ETI or ECMO were evaluated using univariate and multivariable logistic regression analyses and expressed in terms of odds ratios (OR) and 95% confidence intervals (CIs). Variables used in the logistic regression analyses, after evaluation for collinearity, included the following demographic and clinical variables: comorbidities expressed as number of concomitant diseases, pneumonia severity evaluated by baseline SO_2_/FIO_2_ ratio, active COVID-19-oriented therapies (i.e., steroids, immunomodulating agents), and time from symptom onset to start of remdesivir treatment. All *p*-values were two-tailed and considered significant when < 0.05. Owing to the descriptive aims of the study, no formal definition of the sample size was made. All analyses were performed using the Statistical Package for Social Sciences 21.0 (SPSS Inc., Chicago, Ill, USA).

## Results

### General characteristics of the study population.

A total of 1004 patients were enrolled in the study (69% male, 35% aged > 70 years). Almost all patients were admitted to Internal Medicine Units from the Emergency Department (*n* = 987, 98.3%). The main demographic and clinical characteristics are shown in Table 1, and outcome measures in Table 2.

**Table 1:** Patients’ demographic and clinical characteristics and laboratory parameters

	**Total number of patients *N* = 1004**
**Demographics**	
Age in years, median (IQR)	64 (56 -75)
Age > 70 years, n (%)	351 (35.0)
Male gender, n (%)	652 (64.9)
**Clinical features**	
SO_2_/FIO_2_ ratio, median (IQR)	3.03 (2.39–3.39)
**Comorbidities**	
Hypertension, n (%)	509 (50.7)
Diabetes, n (%)	203 (20.2)
Obesity (BMI > 30 kg/m^2^), n (%)	193 (19.2)
COPD, n (%)	116 (11.6)
Neurologic disease, n (%)	114 (11.3)
Coronary heart disease, n (%)	98 (9.8)
Endocrine disease, n (%)	91 (9.0)
Gastrointestinal disease, n (%)	81 (8.0)
Atrial fibrillation, n (%)	71 (7.0)
Cancer, n (%)	64 (6.4)
Stroke/TIA, n (%)	47 (4.7)
Rheumatological disease, n (%)	35 (3.5)
Number of comorbidities ≥ 4, n (%)	172 (17.1)
**Clinical parameters**	
Systolic blood pressure (mean ± SD—mmHg)	128 (± 18.0)
Diastolic blood pressure (mean ± SD—mmHg)	74.7 (± 10.8)
Heart rate (mean ± SD – beats/min)	81 (± 14)
Respiratory rate (mean ± SD – acts/min)	20 (± 6)
**Main laboratory findings**	
ALT, IU/L (mean ± SD)	35 (± 23)
Alkaline phosphatase, IU/L (mean ± SD)	68.1 (± 33.8)
Bilirubin, mg/dL or mmol/L (mean ± SD)	0.6 (± 0.37)
Creatinine, mg/dL (mean ± SD)	0.9 (± 0.28)
eGFR mL/min (mean ± SD)	79.9 (± 25.7)
INR, median (IQR)	1.07 (0.16)
D-Dimer, ng/mL (mean ± SD)	882 (± 1196)
**Selected concomitant therapies at admission**	
ACE-inhibitors/ARBs, n (%)	278 (27.7)
Antiplatelet therapy, n (%)	153 (15.2)
Anticoagulant therapy, n (%)	937 (93.3)
- Low molecular weight heparin (LMWH)	845 (90.1)
- Fondaparinux	60 (6.4)
- Direct oral anticoagulants	21 (2.2)
- Warfarin	11 (1.2)
Antidiabetic therapy, n (%)	247 (24.6)
- Insulin	206 (20.5)
- Metformin	34 (13.8)
- GLP-1 receptor agonist	3 (1.2)
- DPP4-inhibitors	2 (0.8)
**Time from symptom onset to first dose of remdesivir**	
Median, days (IQR)	5 (2–7)
< 3 days, n (%)	400 (38.8)
< 5 days, n (%)	609 (60.6)
< 10 days, n (%)	1002 (99.8)

*SD* Standard deviation, *IQR* Interquartile range, *SO*_*2*_*/FIO*_*2*_ Saturation index, *BMI* Body mass index, *COPD* Chronic obstructive pulmonary disease, *TIA* Transient ischemic attack, *ALT* Alanine aminotransferase, *eGFR* Estimated glomerular filtration rate, *INR* International normalized ratio, *ACE* Angiotensin converting enzyme, *ARBs* angiotensin receptor blockers, *GLP-1* Glucagon-like peptide 1, *DPP4* Dypeptidil-peptidase-4

**Table 2:** Outcome measures in the study cohort

**Duration of remdesivir treatment**	**Total number of patients *N* = 1004**
Median, days (IQR)	4 (4–7)
**Outcome measures**	
Length of hospitalization in days, median (IQR)	10 (7–16)
In-hospital death, n (%)	80 (8.0)
Endotracheal intubation and/or ECMO, n (%)	50 (5)
In-hospital death and/or endotracheal intubation or ECMO, n (%)	114 (11.4)

*SD* Standard deviation, *IQR* Interquartile range, *ECMO* Extracorporeal membrane oxygenation

Of note, the prevalence of comorbidities was high, with 17% of the cohort having ≥ 4 concomitant diseases other than COVID-19. Arterial hypertension (50.7%), diabetes (20.2%), obesity (19.2%), COPD (11.6%), neurologic disease (11.3%) and coronary heart disease (9.8%) were the most relevant comorbidities. The median (IQR) saturation index (SO2/FiO2 ratio) at the time of admission to the Internal Medicine Unit was 3.03 (2.39–3.39), indicating moderate-to-severe respiratory failure. It is worth noting that no difference was found between the severity of respiratory failure at baseline, as assessed by SO2/FiO2 ratio, and the timing of remdesivir administration (*p* = 0.49). Nearly all patients (96.6%) were on oxygen therapy with FiO2 < 60% when they received their first dose of remdesivir.

The median duration of treatment with remdesivir was 4 (IQR 4–7) days. All patients enrolled in the study experienced symptom onset less than 10 days before starting remdesivir; of note, the time from symptom onset to the first dose of remdesivir was ≤ 3 days for 39.8% (400/1004) of the cohort, and ≤ 5 days in 60.6% (609/1004) of cases. Two patients who acquired SARS-CoV-2 infection during hospitalization subsequently developed pneumonia and received early treatment with remdesivir. When considering other therapies oriented to COVID-19 treatments, the great majority of patients (94.8%) were receiving steroids, at a dose of dexamethasone ≥ 6 mg per day (or an equivalent dose of other steroids); immunomodulating agents were used in 68 patients (6.8%), baricitinib being the most frequently prescribed (*n* = 54, 5.4%), followed by tocilizumab (*n* = 14, 1.4%). Anticoagulant treatment was administered in most patients (*n* = 937, 93.3%): low molecular weight heparin (LMWH) was the most commonly used anticoagulant (*n* = 845, 84.2%), followed by fondaparinux (*n* = 60, 6.0%). Other patients (*n* = 21, 2.1%) were on treatment with direct oral anticoagulants (8 patients with rivaroxaban, 8 with apixaban, 3 with edoxaban, 2 with dabigatran), while 11 patients were on warfarin treatment. Only a minority of patients were vaccinated against COVID-19 (*n* = 43, 4.6%).

The main laboratory findings of the cohort are shown in Table 1.

### Outcome measures and risk factors for in-hospital adverse events

The median (IQR) duration of treatment with remdesivir was 4 (4–7) days. The median length of hospital stay was 10 (7–16) days (Table 2).

In-hospital death occurred in 80 patients (8.0%), while the overall total for the composite endpoint of in-hospital death and/or ETI or ECMO was 114 (11.4%). Almost all deaths (74/80, 92.5%) were attributable to COVID-19 pneumonia, while in 6 patients the causes were considered related to conditions other than SARS-CoV-2 infection: sepsis (4 patients), congestive heart failure (1 patient), and lung cancer (1 patient).

No significant difference in length of hospitalization, in-hospital death, or the composite outcome of in-hospital death and/or ETI or ECMO was observed when considering the different time intervals between symptom onset and the first dose of remdesivir (Table 3).

**Table 3:** In-hospital outcome in relation to time from symptom onset to start of remdesivir treatment

**Time from initial symptoms of SARS-CoV-2 infection to first dose of remdesivir**	** < 3 days *n* = 400**	** > 3 days *n* = 604**	***p***	** < 5 days *n* = 609**	** > 5 days *n* = 395**	***p***
Length of hospitalization in days, mean (± SD)	13.8 (± 10.1)	12.7 (± 9.4)	0.059	13.4 (± 9.9)	12.7 (± 9.5)	0.232
In-hospital mortality, n (%)	34 (8.2)	46 (7.6)	0.721	51 (8.2)	29 (7.5)	0.719
Composite outcome (in-hospital mortality and/or ETI or ECMO), n (%)	50 (12.2)	64 (10.4)	0.413	77 (12.6)	37 (9.3)	0.081

*SD* Standard deviation, *ETI* Endotracheal intubation, *ECMO* Extracorporeal membrane oxygenation

Several variables were included in the univariate and multivariable logistic regression analyses model; at multivariable analysis, age, presence of > 4 comorbidities and severity of respiratory failure at baseline as expressed by the SO_2_/FIO_2_ ratio were independently associated with adverse in-hospital outcome, whether considering death alone or the combination of death and/or ETI/ECMO (Tables 4 and 5).

**Table 4:** Risk factors for in-hospital mortality, as identified by univariate and multivariable analyses

				**Univariate analysis**			**Multivariable analysis**		
	**Total 1004**	**Dead *n* = 80**	**Alive *n* = 924**	**OR**	**95% CI**	***p***	**OR**	**95% CI**	***p***
**Symptom onset** ≤ **5 days before first dose of remdesivir**	609 (60.6%)	50 (62.5%)	559 (60.5%)	1.11	0.69–1.78	0.719			
**Comorbidities ≥ 4**	172 (17.1%)	27 (33.8%)	145 (15.7%)	2.77	1.77–20.6	< 0.0001	1.98	1.16–3.38	0.012
**Age > 70 years**	351 (35%)	56 (70%)	295 (31.9%)	5.10	3.02–8.19	< 0.0001	4.31	2.56–7.27	< 0.0001
**SO**_**2**_**/FiO**_**2**_** on admission (mean ± SD)**	2.89 ± 0.85	2.47 ± 0.87	2.92 ± 0.83	0.55	0.43–0.72	< 0.0001	0.54	0.41–0.70	< 0.0001
**Immunomodulating agents**	68 (6.8%)	4 (5%)	64 (6.9%)	0.79	0.49–1.27	0.811			
**ACE-inhibitors/ARBs**	340 (33.9%)	31 (38.7%)	309 (33.4%)	1.43	0.96–2.13	0.091			
**Obesity (BMI > 30)**	193 (19.2%)	16 (20%)	177 (19.2%)	0.95	0.53–1.68	0.882			

*SO*_*2*_*/FIO*_*2*_ Saturation index, *ACE* Angiotensin converting enzyme, *ARBs* Angiotensin receptor blockers, *BMI* Body mass index

**Table 5:** Risk factors for the composite outcome of in-hospital mortality and/or endotracheal intubation (ETI) or extracorporeal membrane oxygenation (ECMO), based on univariate and multivariable analyses

				**Univariate analysis**			**Multivariable analysis**		
	**Total 1004**	**Dead /ETI/ ECMO *n* = 114**	**Alive *n* = 890**	**OR**	**95% CI**	***p***	**OR**	**95% CI**	***p***
**Symptom onset ≤ 5 days before first dose of remdesivir**	609 (60.6%)	77 (67.5%)	532 (59.8%)	0.68	0.45–1.04	0.081			
**Comorbidities ≥ 4**	172 (17.1%)	34 (29.8%)	138 (15.5%)	2.32	1.49–3.59	< 0.0001	1.94	1.19–3.14	0.008
**Age > 70 years**	351 (35%)	66 (57.9%)	285 (32%)	2.92	1.96–4.34	< 0.0001	2.63	1.71–4.04	< 0.0001
**SO2/FiO2 on admission (mean ± SD)**	2.89 ± 0.85	2.34 ± 0.90	2.96 ± 0.81	0.45	0.35–0.56	< 0.0001	0.43	0.34–0.55	< 0.0001
**Immunomodulatig agents**	68 (6.8%)	6 (6.2%)	62 (7.0%)	0.76	0.32–1.82	0.688			
**ACE-inhibitors / ARBs**	340 (33.9%)	43 (37.7%)	297 (33.4%)	1.43	0.96–2.13	0.092			
**Obesity (BMI > 30)**	193 (19.2%)	27 (23.7%)	166 (18.7%)	1.35	0.85–2.15	0.207			

*SO*_*2*_*/FIO*_*2*_ Saturation index, *ACE* Angiotensin converting enzyme, *ARBs* Angiotensin receptor blockers, *BMI* Body mass index

Adverse drug reactions (ADRs), recorded to evaluate the safety profile of remdesivir, were reported in 22 patients (2.2%): in practically all cases, these were not serious (21/22 patients, 95.5%). The most frequent ADRs were: bradycardia (9 patients, including 1 serious non-fatal event) and nausea/vomiting (8 patients). The most common laboratory abnormality was the elevation of alanine aminotransferase above the upper reference level in 7 patients. In 9 patients, remdesivir was discontinued, following persistent nausea and vomiting (3 patients) and persistent bradycardia (6 patients) attributed to the drug; in 2 patients, remdesivir was temporarily discontinued (for one day) due to prescribing errors.

## Discussion

More than 1000 patients hospitalized for COVID-19 pneumonia were enrolled in this observational study between September 2020 and September 2021. This period coincided with the second and third waves of the pandemic, when the wild-type, Alpha and Delta variants of SARS-CoV-2 were predominant, and vaccines were initially being developed and then coming into increasingly widespread use. We focused our attention on patients hospitalized in the real-life, non-critical care setting of Internal Medicine Units. This is of particular significance, since in Italy nearly 70% of patients hospitalized for COVID-19 were managed by Internal Medicine physicians in dedicated COVID-19 units [23, 24].

After many initial failures of drugs thought to be potentially effective (albeit not based on sound, systematic evidence) [2], a global call to find effective antiviral treatments for SARS-CoV-2 infection achieved its first success with the authorization by regulatory agencies of the anti-nucleotide pro-drug remdesivir. Approval for its clinical use (July 2020) by the European Medicines Agency (EMA), and soon after by the Italian Medicines Agency (AIFA), followed the results of randomized placebo-controlled clinical trials: these showed remdesivir to be effective, compared to the best supportive care, in promoting clinical improvement and reducing the need for oxygen support in patients with mild-to-moderate COVID-19 receiving supplemental oxygen [10].

In assessing and contextualizing the results of the present retrospective study, a clear limitation to any evidence-based conclusions regarding the efficacy of the treatment concerned is that the sample included only patients with COVID-19 receiving remdesivir, without a control group. Several descriptive findings can nevertheless be identified.

The first observation springing from this study is that, in a real-world setting more complex than that of a controlled trial in terms of major variables such as comorbidities, remdesivir is a safe drug for the treatment of hospitalized patients with moderate COVID-19. In this study, ADRs were reported in 22 patients (2.2%), showing a very favorable safety profile of remdesivir. While it is important to acknowledge possible bias in reporting ADRs when collecting data retrospectively, their very low incidence here was consistent with that reported in prospective clinical trials [9–14, 25–27] and with the perception of safety acquired from daily clinical experience of the drug.

In this study, the sample differed substantially from those commonly found in RCTs: 35% of the patients enrolled were more than 70 years of age; in addition, the number of comorbidities was high (17.1% of patients with ≥ 4 concomitant diseases). These characteristics mean that our sample of COVID-19 pneumonia patients was at considerably higher risk of progression and complications than is the case for the generally younger and less complex patients included in controlled clinical trials [9–14]. The marked complexity of patients included in the FADOI-RECOVER study, which is the largest retrospective study in Internal Medicine Units on remdesivir to date, affords a particularly relevant viewpoint on the safety of remdesivir in a real-world setting.

Interestingly, the time span between the onset of symptoms and the first dose of remdesivir had no impact when considering in-hospital death or death/ETI as outcome measures. In our cohort, patients mostly received remdesivir early, in almost two thirds of cases within 5 days. Specifically, we did not find any difference in these endpoints when considering patients who received remdesivir treatment more or less than 5 days from symptom onset, or even for those treated within 3 days of their first symptoms. This is consistent with existing reports that the benefits of remdesivir treatment are confined to those patients treated within 10 days of their first symptoms, with little evidence of incremental benefits when remdesivir is administered earlier [14–18, 25–27].

Multivariable analysis identified three variables as significantly affecting mortality and disease progression (evaluated by a step-up in care, such as need for ETI/ECMO): advanced age, burden of comorbidities, and severity of respiratory failure evaluated by baseline saturation index (SaO_2_/FiO_2_).

Overall, this study’s main limitations are related to its retrospective design and the absence of a control group, which restricted efficacy data on remdesivir. The study’s retrospective nature could also have entailed underreporting of ADRs and a degree of incompleteness in data entries provided by patients. While allowances must be made for this, the percentage of ADRs reported is nevertheless low and consistent with the excellent safety profile reported for remdesivir in clinical trials [9–20, 25–27]. Another possible limitation of the study is relative to the timing of remdesivir initiation in relation to symptoms’ onset. We are aware that exact timing of symptoms’ onset in respiratory viral infections can be difficult to assess [28], especially when dealing with retrospective data collection, and these factors should be considered when contextualizing outcome measures in relation to the time span between symptom onset and the start of remdesivir treatment.

By contrast, the great strength of this study is that it provides information on the use of remdesivir in real-world hospital settings: this demonstrates adherence to appropriate use in clinical practice, with benefits in terms of tolerability and safety. More studies gathering real-world data are needed to corroborate these observations – where possible, with control groups so as to shed light on the efficacy of remdesivir in treatment of moderate-to-severe COVID-19 pneumonia in settings other than controlled clinical trials.

In conclusion, in this retrospective study on treatment of moderate-to-severe COVID-19 pneumonia in a real-life context, remdesivir demonstrated an excellent safety profile and tolerability. With remdesivir first administered less than 10 days after the onset of SARS-CoV-2 symptoms in all patients, the timing of the initial dose within this window did not affect hard clinical endpoints such as mortality and the need for mechanical ventilation. Age, severity of disease, and the number of comorbidities were independent predictors of poor in-hospital outcome.

## Data Availability

The datasets used and/or analyzed during the current study are available from the corresponding Author on reasonable request.

## Abbreviations

WHO: World Health Organization
EMA: European Medicines Agency
ADRs: Adverse drug reactions
ATP: Adenosine triphosphate
RNA: Ribonucleic acid
RCTs: Randomized clinical trials
ECMO: Extra Corporeal Membrane Oxygenation
FADOI: Italian Scientific Society of Internal Medicine
RT-PCR: Reverse transcription polymerase chain reaction
AIFA: Italian Medicines Agency
COPD: Chronic obstructive pulmonary disease
eGFR: Estimated glomerular filtration rate
ALT: Alanine aminotransferase
INR: International Normalized Ratio
ARDS: Acute respiratory distress syndrome
ETI: Endotracheal intubation
SD: Standard deviation
IQR: Interquartile range
LMWH: Low molecular weight heparin
EMA: European Medicines Agency
